# Response: Commentary: Totality of the Evidence Suggests Prenatal Cannabis Exposure Does Not Lead to Cognitive Impairments: A Systematic and Critical Review

**DOI:** 10.3389/fpsyg.2020.583516

**Published:** 2020-11-12

**Authors:** Ciara A. Torres, Christopher Medina-Kirchner, Kate Y. O'Malley, Carl L. Hart

**Affiliations:** ^1^School of Social Work, Columbia University, New York, NY, United States; ^2^Department of Psychology, Columbia University, New York, NY, United States; ^3^Division on Substance Use, Department of Psychiatry, New York State Psychiatric Institute, New York, NY, United States; ^4^Department of Psychological Sciences, Swinburne University, Hawthorn, VIC, Australia

**Keywords:** marijuana, prenatal, cognition, impairment, normative data

We appreciate the expressed interest of Chaput et al. (2020) in our recent critical review of the literature investigating the impact of prenatal cannabis exposure on subsequent cognitive functioning of offspring (Torres et al., 2020). We concluded that the available empirical “evidence does not suggest that prenatal cannabis exposure alone is associated with clinically significant cognitive functioning impairments.”

Chaput et al. asserted that we interpreted our cognitive findings as evidence that prenatal cannabis use is safe in this domain. This is not only untrue but also is a misrepresentation of our meticulously cautious interpretations of the current published literature. The words “safe” or “safety” do not appear anywhere in our article, but they appear at least six times in the critique by Chaput et al. Relatedly, the critique takes exception to our manuscript's title—Totality of the Evidence Suggests Prenatal Cannabis Exposure Does Not Lead to Cognitive Impairments: A Systematic and Critical Review—because, they claim it “implies evidence for the safety of prenatal cannabis use…” We disagree. A good title should concisely summarize what was done and what was found (American Psychological Association, 2020). Our title reflects this fundamental guideline. It is also in line with one of the main points that we emphasized in our article: the need for precise language in the prenatal cannabis exposure literature. The word “suggests” is included in our title, but it does not appear in Chaput et al.'s critique. This omission attempts to facilitate the arguments put forth in the critique. Yet, it is both misleading and ignores our recognition of the caveats associated with the current literature.

Surprisingly, Chaput et al. take issue with our efforts to determine the extent to which cognitive performance was truly impaired by comparing research participant's scores against a normative database. The importance of interpreting cognitive findings within the range of performance for age- and education-matched controls cannot be overstated (Hart et al., 2012). This strategy allows researchers and interested parties to determine the clinical relevance, or everyday import, of cognitive findings. What's more, it is a cardinal feature of clinical neuropsychological assessments (Harvey, 2012). Because investigators of previous research did not report individual scores, we urged researchers of future studies to include this information so that each data point can be compared with appropriate norms.

Appropriately, we highlighted limitations that plagued previous studies as well as those associated with our review. Furthermore, we expressed a clear understanding that future studies in this area “may yield a more concerning pattern of effects.” This recognition, however, does not preclude us from drawing data-driven conclusions about the state of the current literature. In fact, the opposite is true. Accordingly, we pointed out that notions asserting prenatal cannabis exposure causes persistent deleterious cognitive effects are not supported by the totality of the current evidence.

Another misapprehension of the critique is that meta-analysis is the only approach from which meaningful conclusions can be drawn. This is patently false. Important and relevant drawbacks of meta-analysis are that it may assume homogeneity of study populations, interventions, controls, and outcomes, and when this assumption is violated by, for example, the inclusion of heterogeneous studies—from high to low quality—results will be biased or incorrect (Greco et al., 2013). Given the amount of heterogeneity among prenatal cannabis exposure studies, we chose to employ an analytic strategy that allowed us to evaluate each study in detail, accounting for limitations and merits, in an effort to decrease the likelihood that real and meaningful effects would be obscured. Although some models can account for heterogeneity, including in study quality, they still do not replace the elegant technique of meticulously reviewing each article for its strengths and limitations. Nor do meta-analyses compare cognitive scores to norms in an effort to determine clinical relevance, as was done in our study. Thus, in our view, this approach is more rigorous than a meta-analysis and the findings yielded reflect an accurate picture of the published evidence.

While we welcome this discussion, the critique does not provide any data or other information that contradicts or invalidates our original conclusions: (1) interpretations of previous findings have frequently overstated the disruptive impact of prenatal cannabis exposure on subsequent cognitive functioning of offspring; and (2) the available empirical database does not support the view that *in utero* cannabis exposure alone causes cognitive impairments in offspring.

## Author Contributions

CT prepared the first draft of manuscript. CM-K, KO'M, and CH revised and edited the manuscript until it was completed. All authors contributed to the article and approved the submitted version.

## Conflict of Interest

The authors declare that the research was conducted in the absence of any commercial or financial relationships that could be construed as a potential conflict of interest.

## References

[B1] American Psychological Association (2020). Publication Manual of the American Psychological Association, 7th Edn. American Psychological Association.

[B2] Chaput K. H. Lebel C. A. McMorris C. A. (2020). Commentary: totality of the evidence suggests prenatal cannabis exposure does not lead to cognitive impairments: a systematic and critical review. Front. Psychol. 11:1891 10.3389/fpsyg.2020.01891 32973603 PMC7471095

[B3] Greco T. Zangrillo A. Biondi-Zoccai G. Landoni G. (2013). Meta-analysis: pitfalls and hints. Heart Lung Vessels 5, 219–225. 24364016 PMC3868184

[B4] Hart C. L. Marvin C. B. Silver R. Smith E. E. (2012). Is cognitive functioning impaired in methamphetamine users? A critical review. Neuropsychopharmacology 37, 586–608. 10.1038/npp.2011.276 22089317 PMC3260986

[B5] Harvey P. D. (2012). Clinical applications of neuropsychological assessment. Dialogues Clin. Neurosci. 14, 91–99. 22577308 10.31887/DCNS.2012.14.1/pharvey PMC3341654

[B6] Torres C. A. Medina-Kirchner C. O'Malley K. Y. Hart C. L. (2020). Totality of the evidence suggests prenatal cannabis exposure does not lead to cognitive impairments: a systematic and critical review. Front. Psychol. 11:816 10.3389/fpsyg.2020.00816 32457680 PMC7225289

